# Oral Vaccination of Grass Carp (*Ctenopharyngodon idella*) with Baculovirus-Expressed Grass Carp Reovirus (GCRV) Proteins Induces Protective Immunity against GCRV Infection

**DOI:** 10.3390/vaccines9010041

**Published:** 2021-01-12

**Authors:** Changyong Mu, Qiwang Zhong, Yan Meng, Yong Zhou, Nan Jiang, Wenzhi Liu, Yiqun Li, Mingyang Xue, Lingbing Zeng, Vikram N. Vakharia, Yuding Fan

**Affiliations:** 1 Yangtze River Fisheries Research Institute, Chinese Academy of Fishery Sciences, Wuhan 430223, China; muchangyong@yfi.ac.cn (C.M.); mengy@yfi.ac.cn (Y.M.); zhouy@yfi.ac.cn (Y.Z.); jn851027@yfi.ac.cn (N.J.); liuwenzhialisa@yfi.ac.cn (W.L.); liyq@yfi.ac.cn (Y.L.); xmy@yfi.ac.cn (M.X.); zlb@yfi.ac.cn (L.Z.); 2 College of Biological Science and Engineering, Jiangxi Agricultural University, Nanchang 330045, China; zhongqiwang@jxau.edu.cn; 3 Institute of Marine and Environmental Technology, University of Maryland Baltimore Country, Baltimore, MD 21202, USA; 4 State Key Laboratory of Developmental Biology of Freshwater Fish, College of Life Sciences, Hunan Normal University, Changsha 410081, China

**Keywords:** grass carp reovirus (GCRV), baculovirus expression, oral vaccine, immune response

## Abstract

The grass carp reovirus (GCRV) causes severe hemorrhagic disease with high mortality and leads to serious economic losses in the grass carp (*Ctenopharyngodon idella*) industry in China. Oral vaccine has been proven to be an effective method to provide protection against fish viruses. In this study, a recombinant baculovirus BmNPV-VP35-VP4 was generated to express VP35 and VP4 proteins from GCRV type Ⅱ via Bac-to-Bac baculovirus expression system. The expression of recombinant VP35-VP4 protein (rVP35-VP4) in *Bombyx mori* embryo cells (BmE) and silkworm pupae was confirmed by Western blotting and immunofluorescence assay (IFA) after infection with BmNPV-VP35-VP4. To vaccinate the grass carp by oral route, the silkworm pupae expressing the rVP35-VP4 proteins were converted into a powder after freeze-drying, added to artificial feed at 5% and fed to grass carp (18 ± 1.5 g) for six weeks, and the immune response and protective efficacy in grass carp after oral vaccination trial was thoroughly investigated. This included blood cell counting and classification, serum antibody titer detection, immune-related gene expression and the relative percent survival rate in immunized grass carp. The results of blood cell counts show that the number of white blood cells in the peripheral blood of immunized grass carp increased significantly from 14 to 28 days post-immunization (dpi). The differential leukocyte count of neutrophils and monocytes were significantly higher than those in the control group at 14 dpi. Additionally, the number of lymphocytes increased significantly and reached a peak at 28 dpi. The serum antibody levels were significantly increased at Day 14 and continued until 42 days post-vaccination. The mRNA expression levels of immune-related genes (IFN-1, TLR22, IL-1β, MHC I, Mx and IgM) were significantly upregulated in liver, spleen, kidney and hindgut after immunization. Four weeks post-immunization, fish were challenged with virulent GCRV by intraperitoneal injection. The results of this challenge study show that orally immunized group exhibited a survival rate of 60% and relative percent survival (RPS) of 56%, whereas the control group had a survival rate of 13% and RPS of 4%. Taken together, our results demonstrate that the silkworm pupae powder containing baculovirus-expressed VP35-VP4 proteins could induce both non-specific and specific immune responses and protect grass carp against GCRV infection, suggesting it could be used as an oral vaccine.

## 1. Introduction

Grass carp (*Ctenopharyngodon idella*) is an economically important freshwater fish, occupying a vital position in aquaculture of China [1]. However, hemorrhagic disease caused by grass carp reovirus (GCRV) poses a serious threat to the grass carp cultivation industry [2], resulting in a greater than 80% mortality rate and significant economic losses [3]. GCRV, a member of the genus *Aquareovirus* in the family *Reoviridae*, was the first viral pathogen identified from aquatic animals in China in 1983 [4]. A comparative study of gene sequences revealed that GCRV could be divided into three distinct genotypes: GCRV I with GCRV-873 as the representative strain, GCRV II with GCRV-HZ08 as the representative strain and GCRV III with GCRV-104 being the only strain found in China [5]. Epidemiological analyses have shown that the three genotypes exist simultaneously, but GCRV II accounts for the major pandemic of grass carp hemorrhagic disease in China [6]. Therefore, it is necessary to find an efficacious novel vaccine for preventing the hemorrhagic disease caused by GCRV II. At present, some vaccines, including inactivated vaccines, attenuated vaccines, recombinant subunit vaccines and DNA vaccines, have been developed and widely used to protect grass carp. However, the variations of different GCRV strains have led to the need of effective subunit vaccines, which can be developed by recombinant techniques [7].

Virus structural proteins often serve as a key antigen capable of stimulating potent immune response against viral infections [8]. To date, several GCRV structural proteins, such as VP35, VP4, VP6 and VP56, have been investigated as the potential subunit vaccine against GCRV infection. For example, GCRV-VP56 expressed in *Escherichia coli* induced significant immuno-protective effects and provided a protective response of about 71–75% for injection–vaccination in grass carp [9]. In another study, Xue et al. (2013) showed that the silkworm pupae powder containing baculovirus-expressed VP6 protein administered orally with feed induced BacFish-vp6 specific antibody in grass carp [10]. VP4 protein is the major outer capsid protein encoded by GCRV segment 6 (S6) and can be used as a candidate subunit vaccine [3], which plays an important role in viral invasion and replication [11,12]. Bioinformatics analysis predicted that the VP35 protein encoded by segment 11 (S11) of GCRV II contained a conserved putative zinc-binding motif CxxC-n16-HxC sequence and was considered to be an outer clamp protein [13]. Liu et al. showed that anti-VP35 serum could effectively neutralize GCRV infection [14] and Gao et al. suggested that recombinant VP35 protein can induce immunity and protect grass carp against GCRV infection [7]. Therefore, the VP35 protein is predicted to be an outer capsid protein and has antigenicity, and hence can be used as a subunit vaccine [7].

The baculovirus expression system has been widely employed as a powerful expression vector for the production of recombinant proteins and development of subunit vaccines in insect cells due to its biological safety, limited replication in insect cells, low cytotoxicity and simplicity of operation [15,16]. Baculoviruses are double-stranded DNA viruses known to infect invertebrates, of which *Autographa californica* nucleopolyhedrovirus (AcMNPV) and *Bombyx mori* nucleopolyhedrovirus (BmNPV) are the most widely studied [17]. Moreover, the silkworm baculovirus expression system has a potential for not only low-cost but also high-capacity production (up to 20% of total cell protein), which is an ideal system for producing vaccines [10].

The oral vaccination route has the advantages of being inexpensive, time-saving and easy to administer without causing any stress to the fish [10,18]. In the present study, we generated a novel recombinant baculovirus BmNPV-VP35-VP4 expressing the recombinant VP35-VP4 protein (rVP35-VP4) of GCRV II and used it to infect silkworm pupae to prepare a lyophilized powder for oral immunity to grass carp. We determined the immune response and further evaluated the protective effects of the recombinant VP35-VP4 protein against GCRV infection in grass carp. These results will help us to understand the immune protective mechanism of viral protein and lay a foundation for the development of oral vaccine for GCRV.

## 2. Materials and Methods

### 2.1. Ethics Statement

The experiment was carried out in strict accordance with the Guide for the Care and Use of Laboratory Animals Monitoring Committee of Hubei Province, China, and the internal protocols (No. YFI2019fanyuding-02) were approved by the Committee on the Ethics of Animal Experiments at the Yangtze River Fisheries Research Institute, Chinese Academy of Fishery Sciences.

### 2.2. Fish, Virus, Cells and Plasmid

Healthy grass carp, with an average weight of 18 ± 1.5 g, were obtained from a farm in Wuhan City (Hubei, China), which were acclimatized to laboratory conditions for two weeks before experimental manipulation. Fish were maintained at 28 °C in aerated water and fed twice a day. From this pool, fish were randomly tested for the presence of GCRV by RT-PCR to ensure that these fish were free of this virus and are not the carriers of GCRV. GCRV-106 strain, used for this study, was isolated from diseased grass carp with severe hemorrhagic disease and identified as GCRV II in our laboratory. *Bombyx mori* embryo cells were generously provided by Dr. Tian Li, Southwest University, China, and maintained at 28 °C in Grace’s Insect Medium containing 10% (*v/v*) FBS (Gibco, Gaithersburg, MD, USA). Cell transfection reagent Cellfectin^TM^ II Reagent was purchased from Thermo Fisher (Waltham, MA, USA). The pFastBac-VP35-T2A-VP4 plasmid was constructed from previous research in our laboratory [19].

### 2.3. Generation of Recombinant Baculovirus

According to the manufacturer’s instructions, the pFastBac-VP35-T2A-VP4 vector was transformed into *E. coli* DH10Bac/BmNPV to generate recombinant Bacmid-VP35-VP4, using the Bac-to-Bac baculovirus expression system (Invitrogen, Carlsbad, CA, USA). The recombinant Bacmid-VP35-VP4 is too large (larger than 135 kb in size) to perform a restriction analysis. Therefore, recombinant Bacmid-VP35-VP4 was confirmed by PCR analysis with the pUC/M13 Forward primer: 5′-CCCAGTCACGACGTTGTAAAACG-3′ and Reverse primer: 5′-AGCGGA TAACAATTTCACACAGG-3′. The Bacmid-VP35-VP4 DNA was transfected into BmE cells to generate the recombinant baculovirus BmNPV-VP35-VP4 with Cellfectin^TM^ II transfection reagent (Invitrogen, Carlsbad, CA, USA). The transfected cells were incubated at 28 °C for 72 h to generate baculovirus and its release into the culture medium. The culture medium was collected and clarified by low-speed centrifugation for 10 min at 1000× *g* as the P1 virus and the virus continuously proliferated through further infection in BmE cells until the P3 viral stock was obtained and kept at 4 °C in the dark. The P1 and P3 viruses were identified by PCR with primer pairs of GCRV-VP35 F: 5′-GGGATCCATGGAACCAGCAAAACCATG-3′ and GCRV-VP4 R: 5′-GGAATTCTCTAGTGATGGTGGTGATGATGAGACGGAGGAGGCCAGTATCGAGTTAATTTGT-3′.

### 2.4. Analysis of VP35-VP4 Expression in BmE Cells by Western Blotting

To confirm that the rVP35-VP4 protein can be expressed in BmE cells, the BmE cells were collected 72 h after infection with BmNPV-VP35-VP4 for SDS-PAGE and Western blot analysis. BmE cells infected with wild-type BmNPV were used as a negative control. The cell extracts were run on 12% SDS-PAGE and the separated proteins were transferred onto PVDF membranes. After blocking with 5% skim milk in phosphate buffered saline (PBS)–Tween (PBST) for 2 h at 37 °C, the membrane was incubated with a mouse anti-His-tag (1:1000 diluted with TBST, Abcam, Cambridge, UK) and the secondary antibody alkaline phosphatase-conjugated goat anti-mouse IgG (1:5000 diluted with TBST, Thermo Fisher, Waltham, MA, USA) and finally visualized by the enhanced chemiluminescence (ECL) method.

### 2.5. Detection of VP35-VP4 Protein Expression in BmE Cells by IFA

To identify the rVP35-VP4 protein expression, BmE cells were infected with recombinant BmNPV-VP35-VP4 in a 12-well microplate and incubated for 4 days. The standard indirect IFA was performed according to the method described in our previous study [19].

### 2.6. Multiplication and Detection of rVP35-VP4 Protein in Silkworm Pupae

To proliferate rVP35-VP4 protein in the silkworm, the silkworm on the first day of 5th instar was inoculated by subcutaneous injection with recombinant BmNPV-VP35-VP4 at amount of about 3 × 10^4^ PFU per silkworm. The silkworm hemolymph was collected at 25 °C for 120 h after infection. The negative control silkworm hemolymph was collected at 72 h post-infection with wild type of BmNPV. All hemolymph samples were subjected to SDS-PAGE and Western blotting according to the method described previously.

### 2.7. Oral Immunization and Sample Collection

Silkworm pupae were collected after 120 h of inoculation with recombinant virus BmNPV-VP35-VP4 to prepare freeze-dried powder and added to the feed at a rate of 5%. The fish feed containing 5% freeze-dried powder was made from pupae of inoculation with wild type of BmNPV as a control. Grass carps were randomly divided into three groups (*n* = 60 for each group). The fish in the vaccination group (VP35-VP4) were fed with the feed containing 5% of freeze-dried powder of the BmNPV-VP35-VP4-infected pupae; the fish in control group (Group CK) were fed with the feed containing 5% of the freeze-dried powder of the wild type of BmNPV-infected pupae; and the fish in normal group (Group N) were fed with the normal dry pellets. The grass carps were fed with prepared feed continuously in accordance with 2% of the fish weight, twice a day (in a final concentration equivalent to 0.02 μg/g (protein per fish)) for 6 weeks and the experimental fish were maintained at 25 ± 2 °C. Thereafter, grass carps in all groups were fed with the normal dry pellets for 4 weeks and the experimental fish were maintained at 25 ± 2 °C. Blood, head kidney, liver, spleen and hindgut tissues were obtained from three fish in each experimental group at 7, 14, 21, 28, 35 and 42 days post-immunization (dpi). All tissues were placed in DEPC-treated homogenate tube, treated with 1 mL TRIzol reagent and stored at −80 °C. Blood sampled from tail venipuncture and the blood cells were counted according to type by using a Neubauer hemocytometer. The blood samples were kept at RT for 2 h to clot before stored at 4 °C overnight, followed by centrifuging at 4 °C, 1500× *g* for 15 min to collect serum and stored at −80 °C. The blood cells were used for the differential leukocyte count of blood smears.

### 2.8. Counting of Blood Cells and Differential Leukocytes

The blood cell counts were done with a 1:200 dilution of the blood sample using Dacie’s reagent and counted in a Neubauer hemocytometer [20]. Additionally, the average of triplicate micro-hematocrits was used to determine the number of red blood cells (RBCs) or white blood cells (WBCs) [21]. Triplicate blood smears for each sample were prepared from fresh blood, then blood smears were air-dried, fixed in methanol, stained with Giemsa for 15 min and washed and dried, and 100 WBCs were randomly counted and classified using oil immersion microscopy [22].

### 2.9. Detection of Serum Antibody Titer by Enzyme-Linked Immunosorbent Assay (ELISA)

According to a previously described method [4], the titer of the antiserum in grass carp was determined by ELISA. Briefly, 100 μL stock solutions containing 5 μg of rVP35-VP4 was coated in each well in a 96-well plate as antigen. After incubation overnight at 4 °C, it was washed with PBST and blocked with 5% skimmed milk powder blocking buffer at 37 °C for 2 h. The plate was washed three times with PBST, and the fish serum (serum was 1:100 dilutions) obtained from the immunized fish at different time points was added to the wells and incubated at 37 °C for 1 h as a primary antibody. The plate was washed again and incubated with 1:1000 diluted horseradish peroxidase (HRP) conjugated rabbit anti-grass carp IgM antibody at 37 °C for 30 min as a secondary antibody. The dilution of the antibody was according to the previous method and product specification [7,9]. Plates were washed again, 0.1 mL 3,3′,5,5′ tetramethylbenzidine (TMB) was added and the color was developed for 30 min at room temperature. The reaction was stopped by adding 100 μL of 2 M sulfuric acid. After calibration with blank control, the OD_450 nm_ of samples was read by automated microtiter plate reader.

### 2.10. Determination of Immune-Related Genes Expression by qRT-PCR

Total RNA was extracted by TRIzol regent (Invitrogen, Carlsbad, CA, USA) in accordance with the manufacturer’s instructions. First strand cDNA was synthesized using the PrimeScript™ 1st strand cDNA Synthesis Kit (TaKaRa, Dalian, China). The expression of genes involved in the immune response (IFN-1, TLR22, IL-1β, MHC I, Mx and IgM) were determined by quantitative real-time polymerase chain reaction (qRT-PCR). The primers for each gene are listed in Table 1. All qRT-PCRs were performed using TB Green^®^ Premix Ex Taq™ II (TaKaRa) and the amplification conditions were carried out as described in our previous study [19]. All samples from the immunized and the control groups were tested in triplicate by using qPCR. The gene expression was normalized using the housekeeping gene β-actin. The relative expression ratios of immune-related genes were analyzed by the 2^−ΔΔCT^ method [23].

### 2.11. Challenge Test

All 30 fish in each group were intraperitoneally injected with 10 μL of 1 × 10^5^ LD_50_ GCRV-106 on 56 days post immunization and then the mortality was recorded daily. The dead fish were collected and RNA from the spleen was extracted to detect the presence of the virus using PCR. The relative percent survival (RPS) was calculated after 14 days of post infection by the following formula: RPS = (1–the ratio of mortality percent in the immunized group to in the control group) × 100%.

### 2.12. Statistical Analysis

The results were expressed as the mean ± standard deviation (SD) and the statistical analysis were carried out with SPSS 20.0 software (SPSS Inc., Chicago, IL, USA). Comparisons between the experimental group and the control group were analyzed by using ANOVA and Tukey’s tests and differences were defined as statistically significant at *p* < 0.05 and extremely significant at *p* < 0.01.

## 3. Results

### 3.1. Generation of Recombinant Baculoviruses in BmE Cells

**Figure 1:**
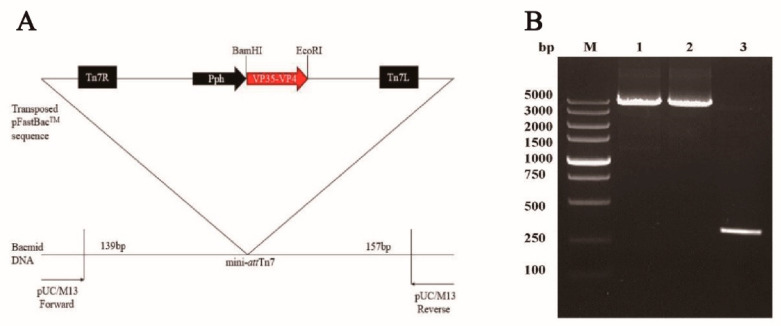
Construction and identification of the recombinant BmBacmid-VP35-VP4: (**A**) schematic construction of the recombinant baculovirus BmNPV-VP35-VP4; and (**B**) PCR amplification of VP35-VP4 from BmBacmid-VP35-VP4 (Lane M, DNA marker; Lanes 1–2, Bacmid-VP35-VP4; Lane 3, Bacmid alone).

**Figure 2:**
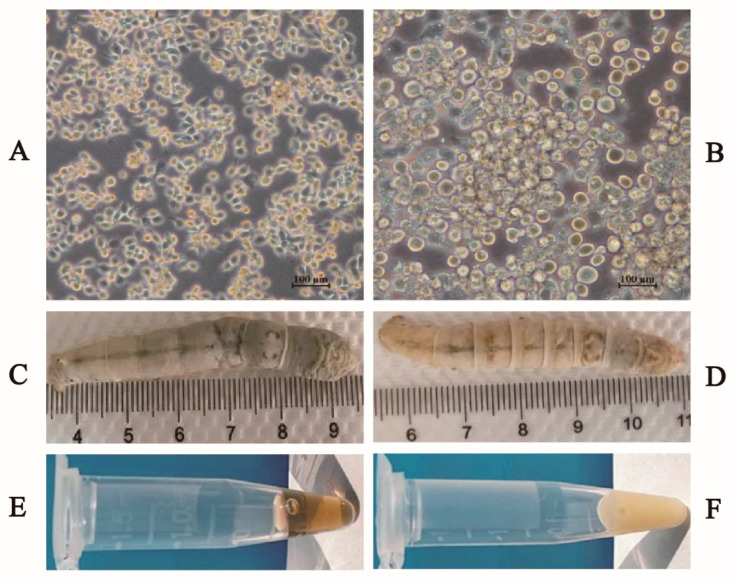
Symptoms of BmE cells and silkworms after transfected/infected with recombinant baculovirus: (**A**) normal BmE cells; (**B**) BmE cells transfected with recombinant BmBacmid-VP35-VP4; (**C**) normal silkworm; (**D**) silkworm infected with rBmBac-VP35-VP4; (**E**) hemolymph of normal silkworm; and (**F**) hemolymph of silkworm infected with rBmBac-VP35-VP4.

**Figure 3:**
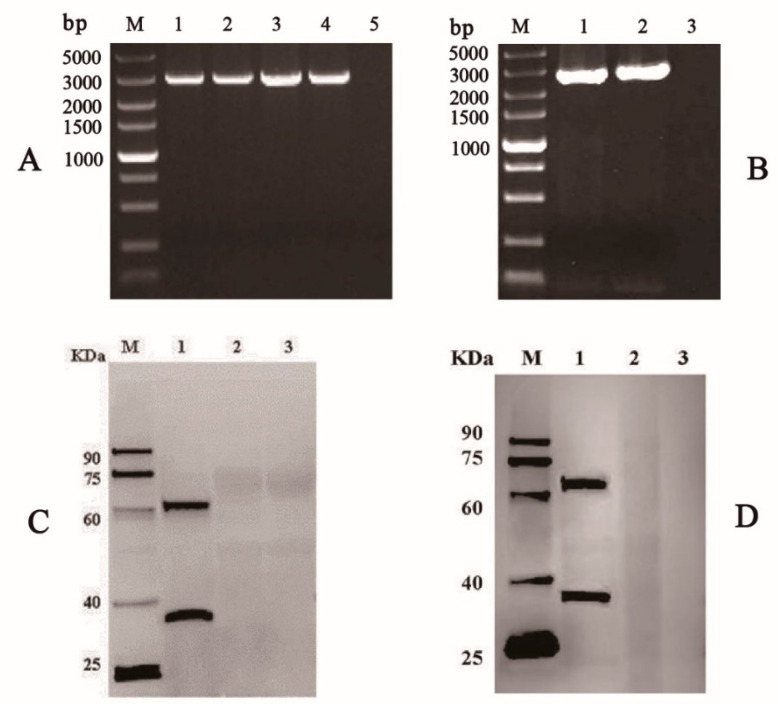
Confirmation and expression analysis of VP35-VP4 in BmE cells and hemolymph of silkworms by PCR and Western blotting. (**A**) PCR amplification of VP35-VP4 from the genome DNA of P1, P2, and P3 recombinant baculoviruses in BmE cells: Lane M, DNA marker; Lanes 1–3, P1–P3 rBmBac-VP35-VP4; Lane 4, rBmBacmid-VP35-VP4; Lane 5, rBmBac-wild. (**B**) PCR amplification of VP35-VP4 in hemolymph of silkworms: Lane M, DNA marker; Lane 1, Hemolymph of silkworms infected with rBmBac-VP35-VP4; Lane 2, rBmBacmid-VP35-VP4; Lane 3, rBmBac-wild. (**C**) (Figure S1) Western blotting analysis of rVP35-VP4 expression in BmE cells: Lane M, protein marker; Lane 1, BmE cells infected with rBmBac-VP35-VP4; Lane 2, BmE cells infected with rBmBac-wild; Lane 3, normal BmE cells. (**D**) (Figure S2) Western blotting analysis of rVP35-VP4 expression in hemolymph of silkworms: Lane M, protein marker; Lane 1, Hemolymph of silkworms infected with rBmBac-VP35-VP4; Lane 2, Hemolymph of silkworms infected with rBmBac-wild; Lane 3, normal hemolymph of silkworms.

To identify the recombinant bacmid, PCR was performed with primers M13 forward and M13 reverse (Figure 1A). A 5.2 kb product was obtained, which was consistent with the theoretical molecular weight detected, suggesting the VP35-VP4 expression cassettes had integrated into the bacmid genomic DNA (Figure 1B). Furthermore, the recombinant plasmid (Bacmid-VP35-VP4) was verified as being correct by sequencing. The purified Bacmid-VP35-VP4 DNA was transfected into BmE cells to obtain recombinant baculovirus, and the transfected BmE cells showed enlarged cell diameter and nuclei sizes, granular appearance, cessation of cell growth, detachment from the flask and cell lysis at three days post-transfection (Figure 2). After three cycles of infection, high titer P3 virus was used as a template and confirmed using PCR with the primers GCRV-VP35/GCRV-VP4 (Figure 3), suggesting that recombinant baculovirus BmNPV-VP35-VP4 was successfully generated.

### 3.2. Expression of VP35-VP4 Protein in the BmE Cells and Silkworm upae

**Figure 4:**
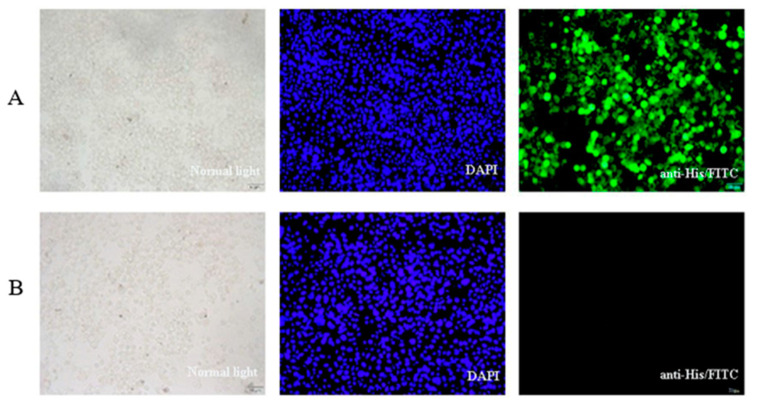
Immunofluorescence assay of recombinant protein expression in BmE cells infected with the recombinant virus: (**A**) BmE cells infected with rBmBac-VP35-VP4; and (**B**) BmE cells infected with rBmBac-wild. From left to right: the BmE cells under normal light, UV light and treated with mouse anti-His-tag and Daylight 488-conjugated goat anti-mouse IgG.

The BmE cells and hemolymph from silkworm infected with P3 baculovirus BmNPV-VP35-VP4 were collected after Day 5 and examined by SDS-PAGE and Western blotting. Two protein bands of around 34 and 67 kDa represent the recombinant VP35 and VP4 proteins, respectively, were visualized by Western blotting, suggesting that VP35-VP4 gene was successfully expressed in the BmE cells and silkworm pupae and two proteins achieve complete cleavage via autocatalytic T2A peptide (Figure 2 and Figure 3). The IFA analysis showed that the VP35-VP4 gene was expressed efficiently in the BmE cells (Figure 4).

### 3.3. Blood Cell Counting

**Figure 5:**
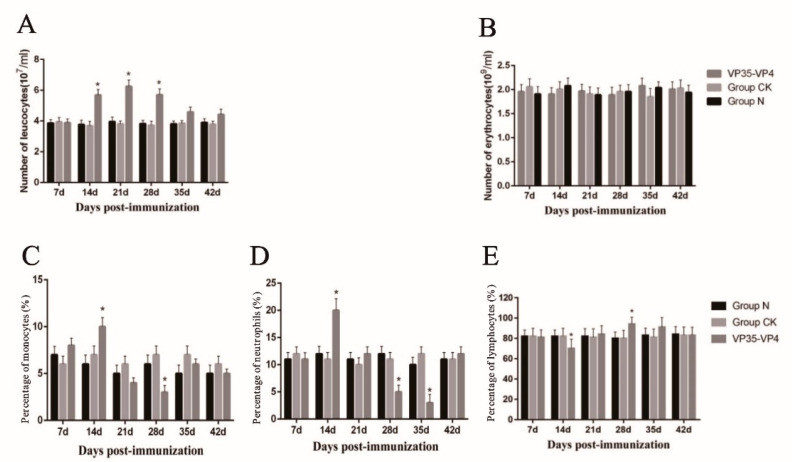
The changes of hematocyte numbers and differential leucocyte count in peripheral blood of grass carp after immunized with oral vaccine. (**A**–**B**) The hematocyte number changes of white blood cells and red blood cells in immunized grass carp, respectively. (**C**–**E**) Differential leucocyte count changes of monocytes, neutrophils and lymphocytes in immunized grass carp, respectively. * significant difference (*p* < 0.05). The significant difference is compared against the group CK group.

Analysis of the hematological parameters indicated that the number of RBCs in the immunized group was 1.96 ± 0.12 × 10^9^/mL and no significant difference compared to the control group. However, the number of WBCs were increased significantly at 14 dpi, significantly higher in immunized fish than in the control group at 14, 21 and 28 dpi (*p* < 0.05) and reached a peak (6.25 ± 0.81 × 10^7^/mL) at 21 dpi (*p* < 0.05) (Figure 5A,B).

### 3.4. Differential Leukocyte Count

Compared to the control group, the percentage of neutrophils and monocytes among the leukocytes in the immunized group increased significantly and reached a peak (19.82 ± 1.18% and 10.12 ± 1.18%) at 14 dpi (*p* < 0.05). The percentage of lymphocyte among the WBCs began to increase and reached the peak of 93.14 ± 0.82% on Day 28 (*p* < 0.05), and it was still higher than the control group at 35 dpi. However, because of the increase in the number of lymphocytes, the percentage of neutrophils and monocytes significantly declined at 28 dpi compared with that of the control group (*p* < 0.05) (Figure 5C–E).

### 3.5. Detection of Serum Antibody Titer by ELISA

**Figure 6:**
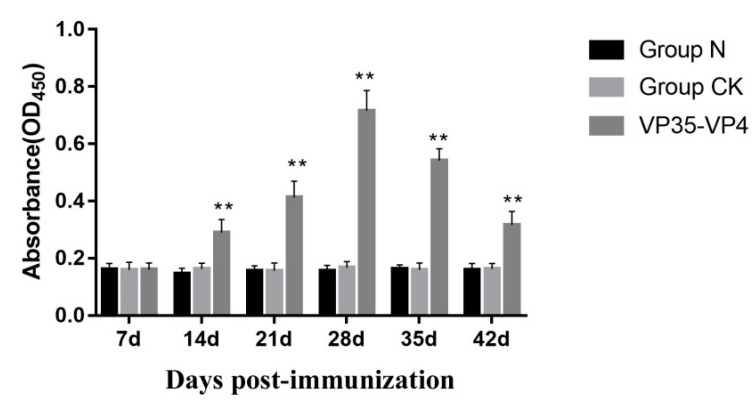
Change of serum IgM antibody levels in grass carp after immunized with oral vaccine. The fish serum was 1:00 dilutions. ** *p* < 0.01. The significant difference is compared against the group CK group.

The results of ELISA show that no specific antibody response was observed in the control group and normal group. However, in the immunized group, specific IgM levels increased significantly at Day 14 (*p* < 0.01) and reached a peak at Day 28 (*p* < 0.01). Subsequently, the antibody levels decreased steadily, but they were still higher than those of the control group at 35 and 42 dpi (*p* < 0.05) (Figure 6).

### 3.6. Expression of Immune-Related Genes

**Figure 7:**
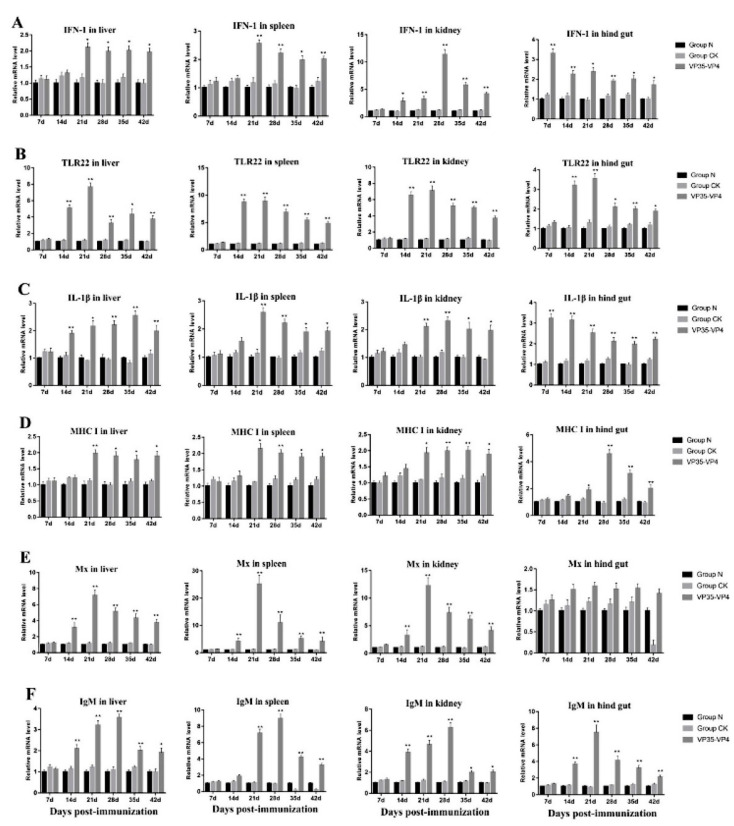
qRT-PCR analysis of the expression of immune-related genes in different tissues of the grass carp on Days 7, 14, 21, 28 and 42 after immunization with oral vaccine: (**A**) IFN-1; (**B**) TLR22; (**C**) IL-1β; (**D**) MHC I (**E**) Mx; and (**F**) IgM. The mRNA level of each gene was normalized on the basis of β-actin gene expression. * *p* < 0.05; ** *p* < 0.01. The significant difference is compared against the group CK group. The relative mRNA expression levels of the immune-related genes were calculated using the 2^−ΔΔCt^ method.

The expression levels of immune-related genes IFN-1, TLR22, IL-1β, MHC I, Mx and IgM were detected in the liver, spleen, kidney and hindgut by qRT-PCR. The transcript levels of the immune-related genes examined increased significantly with different degrees in the immunized group (VP35-VP4), but no significant differences were detected between the control group (Group CK) and the normal group (Group N) (Figure 7). IFN-1 transcription in the spleen and liver began to increase and reached a peak (*p* < 0.05) at 21 dpi. Significant upregulation was observed at 28, 35 and 42 dpi (*p* < 0.05) compared with the control group (Figure 7A). The expression level of the IFN-1 gene in the kidney and hindgut tissue began to increase and reached a peak at 28 and 7 dpi (*p* < 0.05), being approximately 12- and 3-fold higher than that of the control, respectively (Figure 7A). The relative expression levels of TLR22 in all examined tissues started to increase significantly at 14 dpi (*p* < 0.01) and reached their peak values at 21 dpi (*p* < 0.01), 3–8 times higher than the control group (Figure 7B). Meanwhile, the mRNA expression of IL-1β and MHC I in liver, spleen, kidney and hindgut was upregulated by 2–5 times compared to the control group (Figure 7C,D). The mRNA expression levels of Mx in liver, spleen and kidney reached a peak at 21 dpi (*p* < 0.01) (Figure 7E), being approximately 7-, 12- and 25-fold higher than the control, respectively, but there were no significant differences in hindgut. Moreover, in the immunized group, the relative mRNA expression levels of IgM started to increase significantly at 14 dpi in liver and hindgut (*p* < 0.01) and reached the highest value at 21 dpi (Figure 7F), approximately 3.1- and 6.5-fold the control, respectively. In kidney and spleen, the IgM expression levels of the immunized group began to rise significantly at 14 and 21 dpi and reached their peak at 28 dpi (*p* < 0.01), which was about 6–9-fold higher than that in the control group.

### 3.7. GCRV Challenge Test

**Figure 8:**
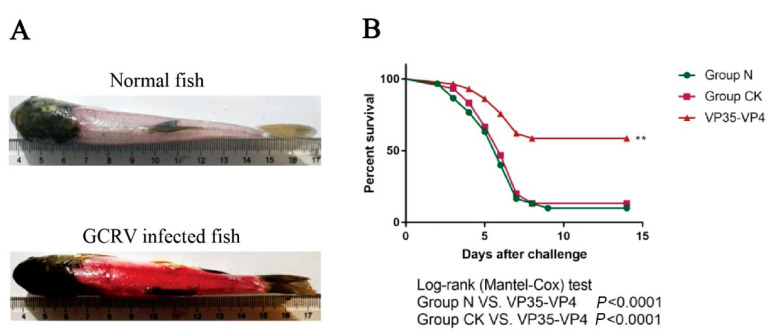
Results of GCRV challenge test. (**A**) Hemorrhagic symptoms induced by GCRV-106. Images show representative fish specimens in the GCRV-106 and control groups, with the latter exhibiting typical muscular hemorrhagic symptoms. (**B**) Cumulative survival percentage of the experimental fish challenged with the virus. ** *p* < 0.01.

After six weeks of continuous oral immunization, all groups of grass carp were challenged with 1 × 10^5^ TCID_50_ GCRV at Week 10 to evaluate the protective effect. Mortality and clinical signs of challenge fish were recorded daily for two weeks after challenge. The results show that the dead fish exhibited typical clinical symptoms of GCRV infection, including varying degrees of hemorrhage at operculum, gill, fin base and muscle (Figure 8A), and GCRV was detected by RT-PCR in dead fish. The challenge test showed that the survival percent in the immunized group was 60% on Day 15 and was significantly higher than that of the normal group (10%) and control group (13%) (*p* < 0.01) (Figure 8B). In addition, the immunized group showed a higher relative survival percent in comparison with that of the control group (56% vs. 4%, *p* < 0.0001).

## 4. Discussion

Baculovirus expression system is a rapid and high yield protein production platform, which has been widely used in basic research and the pharmaceutical industry due to its advantages such as high expression, eukaryotic post-translational modifications and self-assembly of the viral capsid protein into virus-like particles (VLPs) [24,25,26]. Liu et al. used the baculovirus expression system to express the VP6 protein of GCRV and showed that the baculovirus-vectored vaccine can induce immune responses in fish and have the potential to be developed as an oral subunit vaccine [27]. Based on bioinformatic analysis and previous reports, the VP35 and VP4 proteins of GCRV II were predicted to encode the outer capsid proteins and could induce immune response in the fish against GCRV infection [8,11,12,28]. In the present study, we used the baculovirus expression system to express the recombinant VP35-VP4 proteins to prepare subunit vaccine and investigated its immune responses and efficacy against GCRV infection in grass carp. In our work, the VP35 and VP4 proteins are linked by self-cut 2A peptide to generate a subunit vaccine construct that expresses two full-length antigens from a single open reading frame. The 2A sequence is an oligopeptide of the picornavirus family and can undergo post-translational self-cleavage [29]. The novel “cleavage” event in 2A peptide sequence enables efficient, stoichiometric production of discrete protein products in a single vector and separation of genes placed between 2A peptide sequences is nearly 100% [30]. In this work, two protein bands can be clearly observed at about 34 and 67 kDa, representing the recombinant VP35 and VP4 proteins, which suggests that VP35 and VP4 proteins were expressed separately in BmN cells and silkworm by the cleavage action of self-cut T2A peptide and have high shear cut efficiency and balanced upstream and downstream expressions. In addition, a large amount of fluorescence was observed by IFA, indicating that the VP35-VP4 protein was efficiently expressed in BmE cells.

In fish immunization, the route of immunization also plays an important role. Oral vaccines have received increasing attention because they are effective and easy to operate by directly feeding vaccines without causing any stress to the fish [27,31]. Oral immunization of grass carp with freeze-dried powder of silkworm pupae containing VP7 protein of GCRV elicited significant immune responses against GCRV [27]. In this study, the protective efficacy in grass carp after oral administration were evaluated based on population of blood cells, IgM antibody titer in the serum, expression levels of immune-related genes and RPS of immunized fish following GCRV challenge.

The number of RBCs showed no significant change compared with the control, whereas the number of WBCs in the peripheral blood of fish immunized increased by 1.4–1.7-fold at 14, 21 and 28 dpi. The percentages of neutrophils and monocytes in the WBCs increased and were significantly higher than those of control group at 14 dpi, while lymphocytes increased significantly at 28 dpi. A similar trend was observed in the Chinese giant salamander that was injected with a BPL-killed iridovirus vaccine and gibel carp vaccination with β-propiolactone-inactivated cyprinid herpesvirus 2 [21,22]. These results suggest that the WBCs play a more important role in the immune response. After immunization, the numbers of neutrophils and monocytes in the WBCs first increase, indicating that neutrophils and monocytes play an important role in the early innate immune response, and then the number of lymphocytes increases, which are thought to be involved in a specific immune response mediated by lymphocytes [24].

Serum antibody titer of immunized fish is one of the important indices for the evaluation of a vaccine and reflects the level of immune protection in fish [7]. Previous study showed that the antibody titers increased dramatically at 14 dpi in grass carp immunized with VP4 protein of GCRV-GD108 [3]. In the present study, the results of ELISA indicate that the IgM antibody titer of the immunized group significantly increased on Day 14 and reached the highest level on Day 28, and it was higher than the control group at the sixth week after immunization, consistent with the mRNA expression changes of IgM detected by qRT-PCR, suggesting that the oral vaccine could effectively activate fish humoral immunity and antigens, and the protective effect continued for more than one month.

The thymus, kidney and spleen are the largest lymphoid organs in teleost, and hindgut was the main position for the intake of antigen in grass carp [32,33,34]. Our results show that the expression levels of TLR22, IFN-1 and Mx were significantly upregulated and peaked at 21 dpi in the liver, spleen and kidney of immunized grass carp. Chen et al. (2018) studied the immune effect of pC-S6 DNA vaccine in grass carp and demonstrated that the mRNA expression levels of IFN-1 and Mx mRNA were significantly upregulated in vaccinated fish [28]. Previous studies showed that the pC-S11 DNA vaccine and recombinant VP35 protein of GCRV could promote induction of IFN-I and TLR22 in grass carp to defend against GCRV infection [7,24]. It had been reported that the MHC I and IL-1β expressions were significantly increased in the grass carp immunized by surface displaying BL21/InpN/vp7 vaccine and VP7 DNA vaccine using the bacterial ghost as delivery vehicles against GCRV [2,35]. Our data show that mRNA expression of IL-1β increased significantly in vaccinated grass carp and no significant difference was found in the control group. After 21 days of oral immunization with grass carp, the significant upregulation of MHCI gene was detected, suggesting that oral vaccine could induce the antiviral adaptive immune response in grass carp. IgM is the major immunoglobulin isotype of fish and regarded as an indicator of specific immune responses of teleost fish [36]. In this study, IgM gene expression level was significantly upregulated in oral vaccinated group, which was consistent with the production of specific serum antibodies, indicating that specific immunity was triggered by oral vaccine. Fan et al. (2013) found that grass carp was immunized with DNA vaccine of GCRV VP6, and the IgM mRNA expression levels were significantly upregulated [37]. Therefore, we speculate that the activation of related signaling pathways in immune organs and tissues of immunized fish would be beneficial in resisting GCRV infection [38].

The challenge test showed that the survival rate was 60% and the relative percent survival was about 56% (*p* < 0.01) compared to the control CK group. Previous studies have shown that grass carp injected intraperitoneally with 100 μL (0.3 mg/L) recombinant VP35 protein (rVP35) and 1, 3 and 5 μg/g (recombinant protein/fish) of rVP4 have relative percentages of survival of 60%, 47% and 82% after being challenged with GCRV, respectively [3,24]. Although our study appeared to have a similar or lower protective efficacy compared with theirs, the amount of protein (0.02 μg/g (recombinant protein/fish)) in our study was much lower than those used in previous studies. Jiang et al. (2019) successfully constructed the *B. subtilis* CotC-VP4 recombinant spores (CotC-VP4 spores), and orally immunized grass carp showed relative percent survival (RPS) of 47% [37]. In our research, the orally immunized group with the recombinant protein VP35-VP4 showed RPS of 56%, which is higher than that in previous study, thus we speculate that it might be that the two outer capsid proteins VP35 and VP4 of GCRV could induce a higher immune response than that with a single protein in grass carp.

## 5. Conclusions

Our results reveal that the baculovirus expression system is very useful to produce the recombinant subunit vaccine VP35-VP4 of GCRV. The recombinant subunit vaccine VP35-VP4 induced not only innate immunity but also humoral and cellular immunity after oral administration in grass carp and provided protection against GCRV infection. In addition, the interaction between VP35 and VP4, as well as other viral proteins, during GCRV invasion need to be further studied.

## Figures and Tables

**Table 1:** Primers used for real-time PCR analysis.

Gene	Primers (5′–3′)	Annealing Temperature (°C)	Product Size (bp)	Accession No.
TLR22	F:CCATCCATTTAACAGGTGCATAC R:CAGCAGATGTGGAAAGAGACC	58	174	JQ670915.1
IL-1 β	F:TGTGACGCTGAGAGACGGAAA R:GAGTTTCAGTGACCTCCTTCAA	60	190	JX014320
IgM	F:GAGGCATCGGAGGCACATTTC R:TTGGGTCTCGCACCATTTTCTC	55	166	DQ417927
Mx1	F:CTGGGGAGGAAGTAAAGTGTTCT R:CAGCATGGATTCTGCCTGG	57	391	HQ245104
IFN-I	F:AAGCAACGAGTCTTTGAGCCT R:GCGTCCTGGAAATGACACCT	58	78	DQ357216
MHC I	F:CCTGGCAGAAAAATGGACAAG R:CCAACAACACCAATGACAATC	56	271	AY391782
β-actin	F:GATGATGAAATTGCCGCACTG R:TGGTCAGCCCGAAACTATC	58	151	M25013

## Data Availability

The authors confirm that the data supporting the findings of this study are available within the article and its supplementary materials.

## Supplementary Materials

The following are available online at https://www.mdpi.com/2076-393X/9/1/41/s1, Figure S1: Figure 3C’S uncropped blots, Figure S2: Figure 3D’S uncropped blotS.
