# Supplementary figures and images for: Oral Vaccination of Grass Carp (Ctenopharyngodon idella) with Baculovirus-Expressed Grass Carp Reovirus (GCRV) Proteins Induces Protective Immunity against GCRV Infection
Source: Vaccines (Basel). 2021 Jan 12;9(1):41. doi: 10.3390/vaccines9010041 (PMC7827918; doi:10.3390/vaccines9010041)

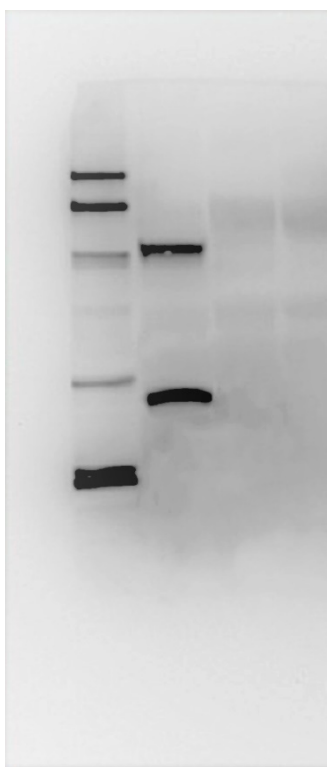

Figure S1 (uncropped blots)

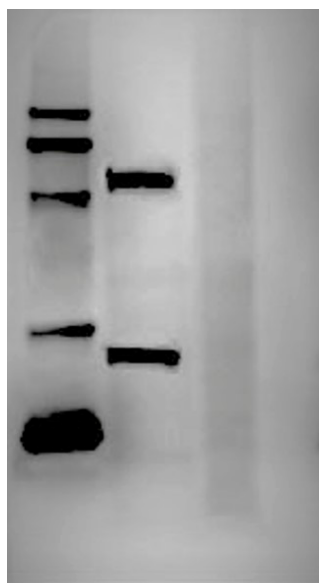

Figure S2 (uncropped blots)

Supplement: Supplementary file 1 [file vaccines-09-00041-s001.pdf]
